# Comparing acute versus AIDS ART initiation on HIV-1 integration sites and clonal expansion

**DOI:** 10.1038/s41392-024-02113-7

**Published:** 2025-01-10

**Authors:** Jun Wang, Nan Xiao, Zhengnong Zhu, Haiyan Qiao, Fang Zhao, Lukun Zhang, Jizhou Gou, Mengji Lu, Yun He, Hongzhou Lu, Qian Li

**Affiliations:** 1 National Clinical Research Center for Infectious Diseases, The Third People’s Hospital of Shenzhen and The Second Affiliated Hospital of Southern University of Science and Technology, Shenzhen, 518112 Guangdong Province China; 2 Clinical Research Center, The Fifth People’s Hospital of Wuxi, Jiangnan University, Wuxi, 214122 Jiangsu Province China; 3 Department of Pathology, Shenzhen Third People’s Hospital, Shenzhen, 518112 Guangdong Province China; 4 Institute of virology, Essen University Hospital, University of Duisburg-Essen, Essen, 45147 Germany

**Keywords:** Infectious diseases, Infection

## Abstract

Early antiretroviral therapy (ART) initiation is known to limit the establishment of the HIV reservoir, with studies suggesting benefits such as a reduced number of infected cells and a smaller latent reservoir. However, the long-term impact of early ART initiation on the dynamics of the infected cell pool remains unclear, and clinical evidence directly comparing proviral integration site counts between early and late ART initiation is limited. In this study, we used Linear Target Amplification-PCR (LTA-PCR) and Next Generation Sequencing to compare unique integration site (UIS) clonal counts between individuals who initiated ART during acute HIV infection stage (Acute-ART group) and those in the AIDS stage (AIDS-ART group). Our analysis revealed distinct clonal distribution patterns, with greater UIS heterogeneity in Acute-ART group and more homogeneity in AIDS-ART group. Monoclonal UIS accumulation, predominantly in-gene regions, was influenced by ART timing and duration, with early treatment delaying this process. Host cell genes integrated by HIV provirus as monoclonal types were enriched in cell cycle and lymphocyte activation pathways. Tumor suppressor genes (TSGs) were more frequently integrated as monoclonal types in AIDS-ART group, suggesting potential risk factors. Overall, we introduced a sequencing method to assess provirus size in human peripheral blood and identified the widespread presence of monoclonal distribution of UIS in AIDS-ART group after long-term treatment. The early intervention helps slow the progress of clonal expansion of infected cells, reducing the formation of stable and persistent reservoirs, and ultimately posing fewer barriers to achieving a functional cure.

## Introduction

Over the past decade, significant efforts have been made to reduce the size of the persistent HIV reservoir and enhance immune control, with the ultimate goal of achieving a functional cure for HIV infection. Despite these efforts, reductions in reservoir size have been modest, and complete eradication remains elusive.^1–5^ Additionally, these interventions have not suppressed viral rebound or significantly lowered viral setpoints after the cessation of therapy, even following decades of rigorous ART.^6,7^ Besides, HIV infection progresses through three stages: Acute HIV Infection, Chronic HIV Infection, and AIDS. Acute infection involves rapid viral replication and flu-like symptoms, while the chronic stage features lower viral levels and asymptomatic persistence; if untreated, it advances to AIDS, characterized by severe immune damage and heightened vulnerability to opportunistic infections.^8^ Early initiation of ART has been shown to improve immune function, reduce mortality, and decrease the size of the HIV reservoir; however, it does not eradicate the virus and often leads to a rapid rebound in viral load following analytical treatment interruption (ATI).^9–12^ Despite these findings, the specific characteristics of HIV reservoirs and proviral genomes in individuals who start ART during the acute stage of HIV infection remain poorly understood, underscoring the need for further research to fully grasp the impact of early ART on the HIV reservoir.

The major obstacle to curing HIV-1 is the persistence of intact, infectious proviruses integrated into the host’s chromosomal DNA, establishing a reservoir of latently infected cells.^13^ This process begins soon after initial infection and, in the absence of ART, results in over 10^8^ new integration events daily, with some persisting for years. During chronic HIV infection, approximately 1 in 100 to 1 in 1000 CD4 + T cells are infected daily, though most newly infected cells die quickly. A small fraction of these cells survives and form detectable clones within weeks, with most carrying defective proviruses (>98%). These cells can nonetheless contribute significantly to viral persistence and produce progeny viruses upon ATI.^14–18^ It is well established that people living with HIV (PLWH) who commence ART early exhibit a smaller HIV reservoir compared to those who start ART later.^11,14,19^ However, the kinetics and characteristics of the viral reservoir, especially in PLWH who begin treatment at the acute stages of infection, remain incompletely understood due to the challenges in obtaining samples and the limitations of current methodologies for detection and analysis during these critical periods.

The distribution of proviruses in individuals on long-term ART is influenced by their initial integration in newly infected cells, and this distribution is further shaped by selection pressures that favor their survival, preferential loss, or clonal expansion.^13,20,21^ Studies have shown that at least 40% of long-lived, persistently infected cells are found within a small number of clones, each originating from a single infected cell. These clonally amplified cells can persist for extended periods, with some documented to last over 11 years. These cells, which share similar UIS, are categorized as monoclonal or oligoclonal types.^22–24^ It is speculated that long-term ART induces the survival of these long-lived amplified clones, which are characterized by deep latency and may have a limited ability to drive rebound viremia upon ATI, posing a significant challenge to HIV eradication. Furthermore, research indicates that proviral integration into chromatin is not-random, favoring certain genes and chromatin regions. HIV-1 DNA preferentially integrates into euchromatin due to its high accessibility. However, under long-term ART, proviruses increasingly target repressive chromatin, specifically heterochromatin, where they gain a selective advantage and persist over time.^6,24–26^ These regions often include genes linked to cancers, supporting the hypothesis that HIV integration into certain genes may promote the proliferation of infected cells, thereby impeding viral decay during ART.^27–30^ However, a detailed assessment of the interplay between the virus, integrated genomic sequences, host cell chromatin, and their impact on gene function and cellular outcomes in PLWH who start ART during the acute phase versus the AIDS phase remains necessary.

This study seeks to address several critical questions regarding the dynamics of HIV reservoirs in the context of ART: (1) How do clonality, homogeneity, and integration sites distribution differ between PLWH who initiate ART during the acute stage versus those who start during the AIDS stage after prolonged infection? (2) Do PLWH initiating ART in the acute stage exhibit distinct patterns of monoclonal UIS accumulation compared to those beginning treatment in the AIDS stage? (3) Are there specific cellular functions associated with HIV integration into genes observed in monoclonal types, potentially supporting the survival and/or proliferation of HIV-infected cells? (4) Which host genes are preferentially targeted for monoclonal integration by HIV proviruses, presenting greater risks for PLWH initiating ART during the AIDS stage compared to the acute phase?

To address these questions, we generated a comprehensive integration sites library using LTA-PCR and Next Generation Sequencing to longitudinally analyze proviral integration sites in HIV-infected individuals. Participants were divided into two groups: those initiating ART during acute HIV infection phase (Acute-ART group) and those beginning ART in the AIDS phase (AIDS-ART group). We examined the distribution of integration sites within the host genome and compared integration patterns of distinct clonal types between these groups. Additionally, we analyzed the biological processes associated with integrated genes, with a particular focus on monoclonal cell amplification and TSGs targeted by HIV provirus integration. These findings offer valuable insights into the dynamics of HIV reservoirs, clarifying mechanisms of clonal expansion at different ART initiation stages and paving the way for targeted strategies to achieve sustained viral suppression and a functional HIV cure.

## Results

### Despite differences in HIV reservoirs and total UIS sequence counts, UIS counts are similar in PLWH initiating ART during AIDS and Acute stage

**Fig. 1:**
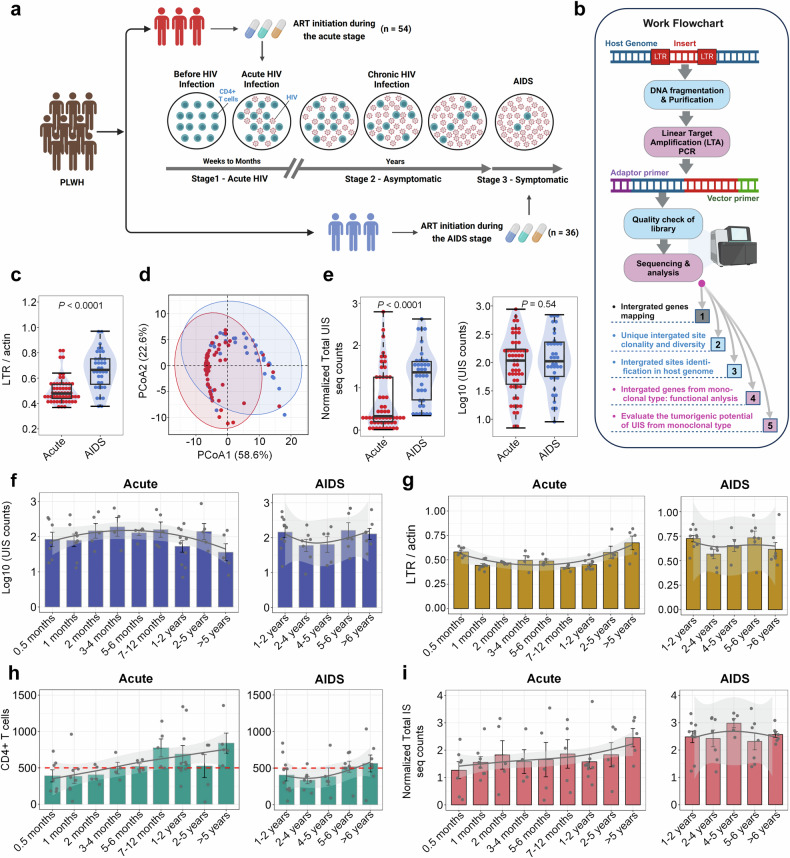
Comparative analysis of HIV total DNA, integration sites, and UIS in Acute-ART vs. AIDS-ART group. **a** Schematic representation of cohort construction and HIV infection stages. This diagram outlines the stages of HIV infection—Acute, Chronic, and AIDS—highlighting the distinct characteristics of each, such as decreased CD4 + T cell counts (dark green circles) and elevated HIV viral replications (magenta dots). The schematic emphasizes the importance of early diagnosis and timely initiation of ART. PLWH are categorized based on the timing of ART initiation: Acute-ART group, refers to PLWH who initiate ART during the acute stage (*n* = 54); AIDS-ART, refers to PLWH who initiate ART during the AIDS stage group (*n* = 36). The image was created using Biorender (https://biorender.com/). **b** Flowchart illustrating the process of provirus integration site sequencing using LTA-PCR and next-generation sequencing, followed by sequencing analysis. Detailed experimental procedures and analytical methodologies are available in the Methods section of the main text and in the Supplementary Files. The image was created using Biorender (https://biorender.com/). **c** Comparison of HIV total DNA levels. Violin plots showing relative gene copies of LTR relative to β-actin (LTR/β-Actin) based on LTR RT-PCR for PLWH who initiated ART during the acute stage (red dot, *n* = 54) versus those who started treatment during the AIDS stage (blue dot, *n* = 36). Wilcoxon test (**d**) PCA plot illustrating the separation of patient groups based on LTR levels relative to glyceraldehyde 3-phosphate dehydrogenase (GAPDH) and β-actin (LTR/GAPDH; LTR/β-actin), along with CD4 + T cell counts. The Acute-ART group (red dots, *n* = 54) and the AIDS-ART group (blue dots, *n* = 36) are distinctly separated along two principal components (Component 1: 58.6%; Component 2: 22.6%), highlighting the differences in these markers that effectively differentiate the two groups of PLWH. **e** Normalized total UIS counts (left panel) and Log_10_-transformed UIS counts (right panel) for the Acute-ART group (red dots, *n* = 54) versus the AIDS-ART group (blue dots, *n* = 36). The y-axis represents total UIS sequence counts normalized to CD4 + T cell percentage (left panel), and the UIS counts, log10-transformed (right panel). **f**, **g** Longitudinal analysis of [Log_10_ UIS] counts and total LTR DNA levels over time in PLWH initiating ART during the acute stage (left panel, *n* = 54) versus those during the AIDS stage (right panel, *n* = 36). The X-axis represents different ART treatment time groups, and the Y-axis represents Log10-transformed UIS counts (**f**) and LTR/Actin counts (**g**), respectively. The gray line indicates the trend line, and the gray shaded area represents the 95% confidence interval (CI) distribution. **h**, **i** Longitudinal analysis of CD4 + T cell counts (**h**) and normalized total IS seq counts (**i**) over time in PLWH initiating ART during the acute stage (left panel, *n* = 54) versus those during the AIDS stage (right panel, *n* = 36). The X-axis represents different ART treatment time groups, and the Y-axis represents CD4 + T cell counts (**h**) and Normalized Total IS (integration site) seq counts (**i**), respectively. The gray line indicates the trend line, and the gray shaded area represents the 95% confidence interval (CI) distribution. (Note: Distinct dots represented as each measurement in each bar chart and data are represented as mean ± SEM; *p* < 0.05 indicates statistical significance; Wilcoxon test for c and e; PERMANOVA test for (**d**) PCoA plot; the terms ‘Acute’ and ‘AIDS’ in all figures refer to PLWH who initiated ART during the acute stage (also referred to as the Acute-ART group) and those who initiated ART during the AIDS stage (also referred to as the AIDS-ART group), respectively)

It’s widely recognized that early access to ART is critical for managing HIV progression and improving long-term outcomes. Figure 1a presents flowcharts for two cohorts: those initiating ART during the acute stage (Acute-ART group) and those initiating ART during the AIDS stage (AIDS-ART group), illustrating their progression through the three stages of HIV infection and the cohort selection process. Figure 1b outlines the key analytical steps conducted in this study following Gene-IS sequencing, with detailed sequencing steps shown in supplementary Fig. 1. Among PLWH, the Acute-ART group exhibits lower viral reservoir levels compared to the AIDS-ART group (Fig. 1c, supplementary Fig. 2a). The principal coordinates analysis (PCoA), based on LTR DNA (normalized by β-actin and GAPDH, respectively) and CD4 + T cell counts, was followed by a PERMANOVA test to assess significant difference between the two groups. The results, with an *R*^2^ of 0.2094 and *p* < 0.0001, show a significant distinction between the Acute-ART and AIDS-ART groups (Fig. 1d).

We further quantified UIS counts,^31^ normalized UIS counts, and total UIS sequence counts relative to CD4 + T cell counts, using log10 transformations. Our analysis shows that total UIS sequence counts, both normalized and unnormalized, were higher in PLWH who initiated ART during the AIDS stage compared to those who started ART during the acute stage (Fig. 1e, left panel; supplementary Fig. 2b, left panel). UIS counts, both normalized and unnormalized, were similar across both groups (Fig. 1e, right panel; supplementary Fig. 2b, right panel). In the Acute-ART group, UIS counts increased with ART duration up to 4 months, but remained lower after 5 years. In contrast, the AIDS-ART group exhibited lower UIS counts during the 2–4 year period, followed by a slight increase thereafter (Fig. 1f). Normalized UIS counts are provided in supplementary Fig. 2c.

Total LTR DNA levels peaked at 0.5 months, declined to their lowest at 7–12 months, slightly rebounded after 5 years in the Acute-ART group, and later stabilized at levels similar to those of the AIDS-ART group (Fig. 1g). CD4 + T cell counts revealed that 26 out of 54 individuals (26/54) in the Acute-ART group had counts above 500 cells/µL, compared to 14 out of 36 individuals (14/36) in the AIDS-ART group (Fig. 1g). Total IS sequencing counts in the Acute-ART group followed a pattern similar to CD4 + T cell counts, with the lowest levels observed at 0.5 months (supplementary Fig. 2d). For normalized total IS counts, a steady increase was seen in the Acute-ART group, while the counts remained consistently higher in the AIDS-ART group (Fig. 1i).

These findings suggest that while total UIS sequence counts and LTR DNA levels differed between the Acute-ART and AIDS-ART groups, UIS counts were similar across both groups. This similarity indicates that, in PLWH who initiated ART during the AIDS stage, certain UIS were associated with a higher number of clonally expanded cells containing integrated proviruses.

### PLWH initiating ART during the AIDS stage exhibit an increased prevalence of monoclonal UIS and reduced homogeneity

**Fig. 2:**
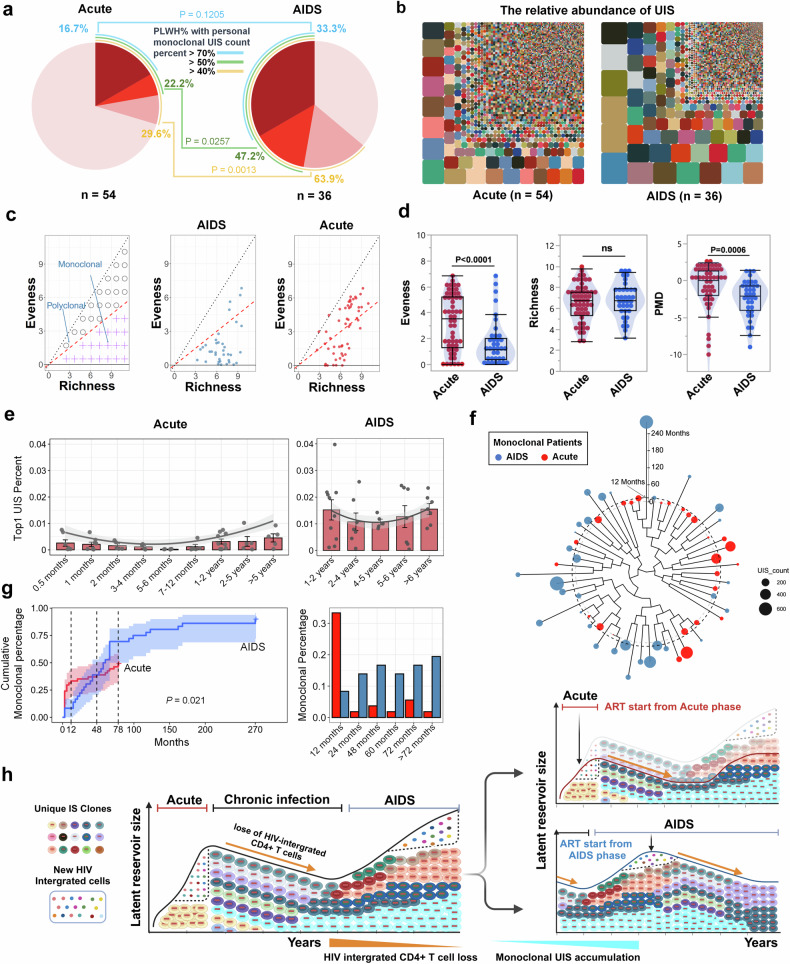
Comparative analysis of UIS clonality in Acute-ART group vs. AIDS-ART group. **a** Pie chart analysis of monoclonal UIS proportions. The pie chart compares the proportion of monoclonal UIS in PLWH who initiated ART during the acute phase (*n* = 54) versus those who began treatment during the AIDS stage (*n* = 36). Colored bands around the outer edge of the pie chart represent the percentage of PLWH with personal monoclonal UIS counts >70% (blue), 50% (green), and 40% (yellow), respectively. Significant differences between groups are indicated by *P*-values by Chi-square test (*P* = 0.1205 for 70%, *P* = 0.0257 for 50%, *P* = 0.0013 for 40%). **b** Mosaic chart of UIS distribution. This mosaic chart shows the relative percentages of the top 5000 UIS in two groups: the Acute-ART group (left, *n* = 54) and the AIDS-ART group (right, n = 36). Larger blocks indicate greater homogeneity in clonal distribution patterns, while a higher number of smaller blocks represents a greater prevalence of monoclonal UIS within the group, and vice versa. **c** PMD analysis. This method assesses UIS clonality based on two dimensions: Richness (variety of UIS) and Evenness (distribution uniformity). The panel categorizes UIS as polyclonal or monoclonal, with the AIDS-ART group (middle) exhibits greater monoclonality, while the Acute-ART group (right) showing higher evenness. **d** Comparative analysis of PMD analysis in Acute-ART group vs. AIDS-ART group. The plot compares the evenness, richness, and PMD data between PLWH initiating ART during the acute stage (red dot) versus those during the AIDS stage (blue dot). **e** Longitudinal analysis of individual Top1 UIS percentage. This panel presents the percentage of the top 1 UIS counts for PLWH initiating ART during the acute stage (left panel) versus those during the AIDS stage (right panel). The *X*-axis represents different ART treatment time periods, and the *Y*-axis shows the top 1 UIS percentage. The gray line indicates the trend, with the gray shaded area representing the 95% confidence interval (CI). **f** Clustering of monoclonal UIS by Evenness, Richness, and ART duration. Patients are clustered based on Evenness and Richness data, with ART duration factored into the analysis. Monoclonal individuals in the AIDS group are shown in blue, while those in the acute group are shown in red. The black dashed line represents the 12 month mark. The bubble sizes represent for the UIS counts. The lager bubble represent for more UIS counts. **g** Cumulative curve of individuals percentage with monoclonal UIS during ART duration. The cumulative curve (left) displays the percentage of individuals with monoclonal UIS over time in the Acute-ART group (red line) versus AIDS-ART group (blue line). The curve shows a rapid increase in the percentage of PLWH initiating ART during the acute stage with monoclonal UIS within the first 12 months, followed by a more moderate rise. In contrast, PLWH initiating ART during the AIDS stage experience a substantial and immediate increase, reaching nearly 78% after 180 months. The bar chart (right) indicates the PLWH’ percentage with monoclonal UIS during different ART time periods, with red bars representing PLWH who initiated ART during the acute stage and blue bars representing those who started ART during the AIDS stage. **h** Schematic representation of proviral integration dynamics. The upper panel illustrates the dynamics of proviral integration across different stages of HIV infection, emphasizing the high rate of new integration events and the expansion of the latent reservoir. The diagram traces the progression from initial infection to detectable clonal expansion, where most newly infected cells perish, but a subset forms detectable clones. During the acute phase, there is increased diversity in provirus-integrated cells and UIS clones. Monoclonal UIS and cell clonality peak towards the end of the acute phase and then decline during the chronic infection stage, accompanied by a loss of HIV-integrated CD4 + T cells, as shown by the black trend line. In the AIDS stage, monoclonal UIS and cell clonality continue to accumulate. The middle panel demonstrates that initiating ART during the acute phase reduces the overall size of the latent reservoir and delays monoclonal UIS accumulation, leading to fewer integration site clones, as indicated by the red trend line. The lower panel shows that PLWH initiating ART during the AIDS stage have a larger latent reservoir and a higher prevalence of monoclonal UIS, along with increased homogeneity of monoclonal integration sites, as indicated by the blue trend line. The image was created using Biorender (https://biorender.com/). (Note: Distinct dots represented as each measurement in each bar chart and data are represented as mean ± SEM; *p* < 0.05 indicates statistical significance; Wilcoxon test for d; random linear regression test for g; the terms ‘Acute’ and ‘AIDS’ in all figures refer to PLWH who initiated ART during the acute stage (also referred to as the Acute-ART group) and those who initiated ART during the AIDS stage (also referred to as the AIDS-ART group), respectively)

To assess the clonality of UIS, we analyzed the percentage of each UIS in PLWH. In the AIDS-ART group, 33% of PLWH had a single UIS that accounted for >70% of the total, compared to 16.7% in the Acute-ART group. When monoclonal UIS were defined as a clonal expansion with a single UIS comprising >50% of the total, 47.2% of PLWH in the AIDS-ART group met this criterion, compared to 22.2% in the Acute-ART group, showing a significant difference (*P* = 0.0257). Similarly, when monoclonal UIS were defined as a single UIS accounting for >40% of the total, 63.9% of PLWH in the AIDS-ART group met this threshold, compared to 29.6% in the Acute-ART group, again showing a significant difference (*P* = 0.0013) (Fig. 2a).

To visualize the overall composition of UIS in both groups, we calculated the percentage of each UIS relative to the total LTR DNA for each individual. Comparing these relative UIS percentages between the two groups (Fig. 2b), we observed a higher prevalence of monoclonal UIS in PLWH who initiated ART during the AIDS stage compared to those who started during the acute stage. This suggests that PLWH initiating ART during the AIDS stage tend to have larger UIS sizes. Notably, distinct clonal distribution patterns were observed based on the stage of ART initiation: PLWH imitating ART during the acute phase exhibited greater heterogeneity, while those initiating ART during the AIDS stage displayed more homogeneity (Fig. 2b).

To further evaluate UIS clonality, we performed a Polyclonal-Monoclonal Distance (PMD) analysis^32^ (Fig. 2c). This approach evaluates the diversity of UIS cells using two key dimensions: (1) Richness, which refers to the variety of UIS present in a sample, indicating the breadth of integration events, and (2) Evenness, which measures the distribution of UIS among cells, reflecting whether certain UIS are disproportionately dominant. Based on these dimensions, we classified the clonality of UIS cells. A sample is considered polyclonal if there is no dominant clonal expansion, characterized by high richness and evenness. In contrast, a sample is considered oligoclonal if dominant cloning occurs, where one or a few UIS predominate, resulting in lower evenness (Fig. 2c, left).

This analysis determines whether IS are broadly distributed among a variety of cells (polyclonal) or concentrated in a few dominant clones (monoclonal) in the two groups (Fig. 2c, middle and right). The results indicate significantly higher evenness in the Acute-ART group, suggesting a more homogeneous distribution of UIS. In contrast, the PMD was significantly lower in the AIDS-ART group, reflecting a greater tendency toward dominant cloning and monoclonality. Despite these differences, there was no significant difference in richness between the two groups, suggesting that the variety of UIS remains similar across both groups (Fig. 2d).

### There is delayed accumulation of monoclonal UIS in PLWH initiating ART during the acute stage compared to the AIDS stage

To assess the changes in monoclonal UIS percentages over different treatment periods, we analyzed the proportion of the top 1 UIS relative to the total LTR DNA in each individual. For those who initiated treatment during the acute phase, the percentage of the top 1 UIS was initially higher at 0.5 months post-treatment. This percentage then decreased to a lower level during the 5–6 month period, then gradually increased again up to 5 years post-treatment. However, it consistently remained lower than the percentage observed in PLWH who initiated ART during the AIDS stage across all time points. These observations suggest that while there is an early spike in monoclonal UIS percentages shortly after treatment initiation in the Acute-ART group, clonality temporarily decreases before rising again over the long term. Yet, it never reaches the high levels observed in the AIDS-ART group, indicating more controlled and less dominant clonal expansion in the Acute-ART group (Fig. 2e).

Next, we clustered all PLWH with monoclonal UIS based on Evenness and Richness data, incorporating ART duration to rank them within the cluster tree. The results indicated that most PLWH who initiated ART during the acute stage developed monoclonal UIS within the first 12 months of treatment, whereas those initiated ART during the AIDS stage typically developed monoclonal UIS after 12 months (Fig. 2f).

To further explore monoclonal UIS accumulation over time in both groups, we plotted a cumulative curve of individuals with disappearing monoclonal UIS during ART duration. This curve highlights the emergence and growth of monoclonal UIS following treatment. By visualizing the cumulative percentage of PLWH with monoclonal UIS over time, we observed that a rapid increase in the acute-ART group within the first 12 months, followed by a more gradual rise. In contrast, the AIDS-ART group experienced a sharp and immediate increase, reaching nearly 78% after 180 months (Fig. 2g, left). The bar chart further illustrates the delayed accumulation of monoclonal UIS in the AIDS-ART group compared to the acute-ART group across different ART time points (Fig. 2g, right).

Overall, these findings suggest that PLWH who initiated ART during the acute stage experience a delay in the accumulation of monoclonal UIS accumulation, whereas those who start ART during the AIDS stage exhibit a rapid and substantial increase in monoclonal UIS over time.

### Monoclonal UIS accumulation is a dynamic process influenced by both ART timing and treatment duration

The accumulation of monoclonal UIS is a gradual and dynamic process that unfolds over several years, with specific clones expanding as certain infected cells persist and proliferate over time.^6^ In the Acute-ART group, UIS Evenness—which reflects diversity and is inversely related to monoclonality—initially increased within the first 12 months of treatment but subsequently decreased with extended treatment duration. Conversely, in the AIDS-ART group, UIS Evenness decreased from the onset of treatment and continued to decline over time (supplementary Fig. 2e). Richness, indicating the emergence of new IS, rose consistently during the early phase of ART initiation in the Acute-ART group before tapering off, while it displayed a continuous increase in the AIDS-ART phase (supplementary Fig. 2f). The longer the duration since initial infection, the more opportunity exists for these dominant clones to establish.

The duration of ART further influences this process. While prolonged ART effectively suppresses new infections, it allows existing infected clones with integrated proviruses to persist and proliferate, gradually dominating the viral reservoir due to their longevity and proliferative capacity. The schematic representation of proviral integration dynamics (Fig. 2h). The upper panel illustrates the trajectory of proviral integration across different stages of HIV infection, emphasizing the high frequency of new integration events and the expansion of the latent reservoir. The diagram traces the progression from initial infection to detectable clonal expansion, where most newly infected cells perish, but a subset forms detectable clones. During the acute phase, there is increased diversity in provirus-integrated cells and UIS clones. Monoclonal UIS and cell clonality reach a peak by the end of the acute phase and then decline during the chronic infection stage, alongside a reduction in HIV-integrated CD4 + T cells, as shown by the black trend line. In the AIDS stage, monoclonal UIS and cell clonality continue to accumulate. The middle panel demonstrates that initiating ART during the acute phase reduces the overall size of the latent reservoir and delays monoclonal UIS accumulation, resulting in fewer integration site clones, as indicated by the red trend line. The lower panel shows that PLWH who initiate ART during the AIDS stage exhibit a larger latent reservoir and a higher prevalence of monoclonal UIS, along with increased homogeneity of monoclonal integration sites, as indicated by the blue trend line.

### Chromatin modification and safe harbor regions are preferred sites for HIV integration during acute phase than AIDS phase but are more prone to loss during treatment

HIV rapidly integrates its DNA into the host genome, with the structure and accessibility of chromatin playing crucial roles in this process. Chromatin regions with specific histone modifications, such as H3K4me3, H3K9ac, are especially preferred for HIV integration due to their open chromatin structure and active transcriptional state. Additionally, safe harbor regions that are active yet less likely to disrupt essential host functions—support stable viral integration. Enhancer regions, which are highly accessible and facilitate recruitment of HIV integration machinery, further promote efficient HIV integration.^27,33,34^

**Fig. 3:**
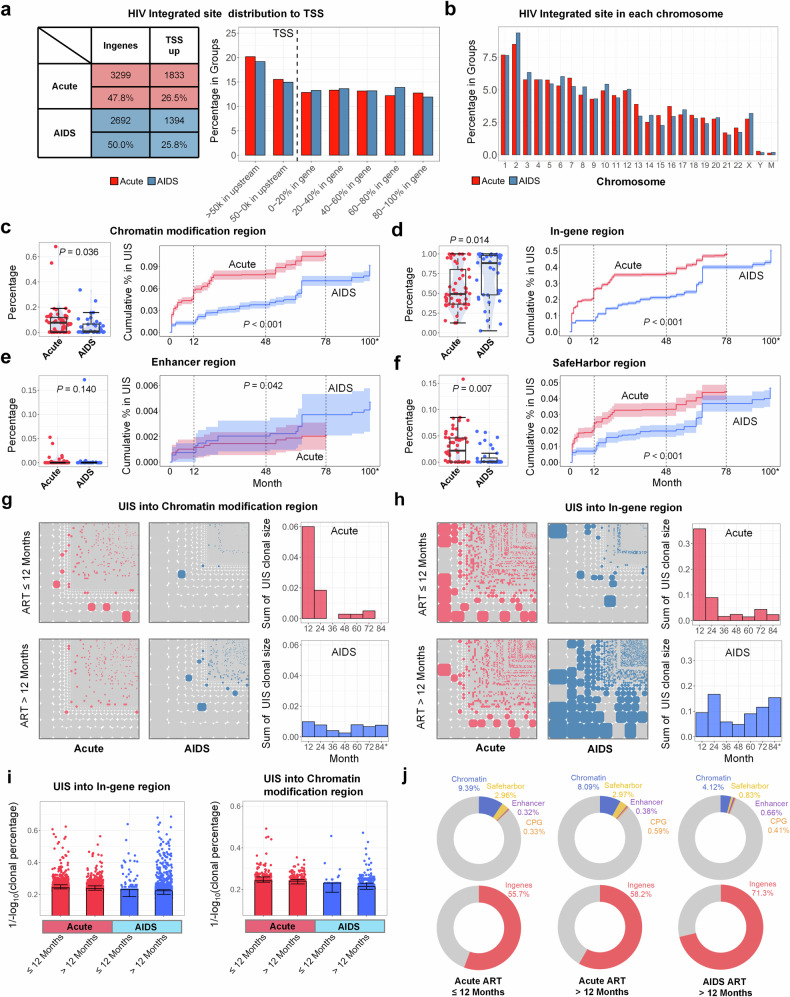
Chromatin modification and safe harbor regions are preferential sites for HIV integration in the Acute-ART group than AIDS-ART group but are more prone to loss during treatment. **a** Distribution of HIV integration across genomic regions. The bar chart compares the frequency of HIV proviral DNA integration into in-gene regions versus upstream promoter regions (TSS up) in PLWH initiating ART during the acute stage (*n* = 54, red) and those in the AIDS stage (*n* = 36, blue). No significant differences are observed in the distribution of integration sites in host genome between the two groups (left). The accompanying table indicates that ART in the acute-phase group, 47.8% (3299) of integrations occur in in-gene regions, and 26.5% (1833) in upstream promoter regions. In contrast, in the AIDS group, 50.0% (2692) of integrations occur in in-gene regions, with 25.8% (1394) in upstream promoter regions (right). **b** Chromosomal distribution of HIV integration sites. The bar chart illustrates the distribution of HIV integration across different chromosomes in both groups, revealing no obvious variation between those initiating ART during the acute phase (red) and the AIDS stage (blue). **c**–**f** Cumulative preference for HIV integrated UIS into chromatin regions over time. The comparison shows the percentage of HIV integration in all patients from two groups: those initiating ART during the acute phase (red) and those in the AIDS stage (blue) (left panels). The cumulative curves illustrate the preference for HIV integration into different genomic regions over time (y-axis representing cumulative % in UIS, x-axis representing months) (right panels). These regions include chromatin modifications (**c**), in-gene regions (**d**), enhancer regions (**e**), and safe harbor regions (**f**). **g**, **h** Mosaic chart of UIS distribution across chromatin modification and in-gene regions among different disease groups. This mosaic chart displays the UIS in Chromatin modification region (**g**) and in-genes region (**h**) in four groups—Acute-initiated ART (≤12 months and >12 months) and AIDS-stage initiated ART (≤12 months and >12 months) (left panel). The bar charts in (**g**) and (**h**) display the total UIS clonal sizes across various time periods (right panel). **i** Normalized and Log-transformed clonal percentage of UIS into region of in-gene regions (left panel) and chromatin modification (right panel) in four groups. **j** Donut charts illustrating HIV integration preferences across different groups. PLWH who initiate ART within the first 12months of the acute stage show a higher number of UIS integrated into chromatin modification regions (blue), and more UIS integrated into safe harbor regions (yellow) and CpG islands (orange) beyond 12 months in Acute-ART group. In contrast, PLWH initiating ART after 12 months during the AIDS stage exhibit fewer of these clonal UIS types, with integration primarily occurring in in-gene regions (red). (Note: Distinct dots represented as each measurement in each bar chart and data are represented as mean ± SEM; *p* < 0.05 indicates statistical significance; Wilcoxon test for (**c**–**f**) left panel; random linear regression test for (**c**–**f**) right panel; the terms ‘Acute’ and ‘AIDS’ in all figures refer to PLWH who initiated ART during the acute stage (also referred to as the Acute-ART group) and those who initiated ART during the AIDS stage (also referred to as the AIDS-ART group), respectively)

When comparing HIV integration patterns between PLWH who initiated ART during the acute stage and the AIDS stage, several key observations were made: (1) Integration into genomic regions: HIV proviral DNA integrates more into in-gene regions of genes than into upstream of TSS promoter regions, with no difference between two groups (Fig. 3a. left panel). (2) Chromosomal Distribution of Integration: The pattern of HIV integration across chromosomes was largely similar between PLWH who initiated ART during the acute stage and those in the AIDS stage, showing no significant differences in chromosomal distribution (Fig. 3b). (3) Preference for chromatin modification and safe harbor regions: In the Acute-ART group, HIV integrates more frequently into regions with active chromatin modifications and within safe harbor regions, compared to the AIDS-ART group. In contrast, PLWH who initiate ART during the AIDS stage showed a greater preference for HIV integration into in-gene regions than those initiating ART during the acute stage (Fig. 3c–f).

(4) Impact of ART on HIV integration and imbalance in UIS accumulation across groups: Fig. 3g illustrates a higher percentage of monoclonal UIS within chromatin modification regions in the Acute-ART group during the first 12 months of treatment, followed by a sharp decline afterward. In contrast, in the AIDS-ART group, the percentage of UIS in chromatin modification regions remains consistently lower over time. Figure 3h shows that in the Acute-ART group, UIS accumulation in in-gene regions is significantly higher during the first 12 months of treatment compared to later stages. After this period, UIS in in-gene regions tend to decrease, whereas UIS in in-gene regions are consistently higher in the AIDS-ART group, except during the first 12 months. Figure 3i presents a comparative statistical analysis of the normalized clonal sizes of UIS in both in-gene and chromatin modification regions, analyzed within and beyond 12 months for both AIDS- and Acute-ART groups. Comparing UIS percentages across groups, UIS in chromatin modification regions is highest in the Acute-ART group within the initial 12 months, whereas UIS in in-gene regions reaches its highest levels in the AIDS-ART group after 12 months (Fig. 3i).

In the supplementary section, we conducted a detailed comparison of chromatin modifications between the Acute-ART and AIDS-ART groups, focusing on markers including CpG, H3K4ac, H3K27ac, H3K4me1, H3K4me3, H3K9me1, H3K27me3, and H3K9me3. The cumulative curve for integration sites within chromatin modification regions (CpG, H3K4, H3K9, H3K27) consistently shows higher values in the Acute-ART group compared to the AIDS-ART group (supplementary Fig. 3a–e). Additionally, in supplementary Fig. 4, analysis of UIS clonal size over the course of ART reveals that UIS within chromatin modification regions had larger clonal sizes in the Acute-ART group compared to the AIDS-ART group, particularly in regions marked by H3K4ac (*p* = 0.012), H3K9me3 and H3K4me3 (*p* = 0.003), and CpG (*p* < 0.001) (supplementary Fig. 4a, b). Among chromatin modification markers, H3K4me3 (27.1%) and H3K4ac (27.1%) were the most prevalent as markers of provirus integration in the Acute-ART group, whereas H3K4ac (31.1%) and H3K27me3 (30.4%) were predominant in the AIDS-ART group (supplementary Fig. 4c). We also conducted a clonal size analysis of UIS in in-gene regions, enhancers, and safe harbor regions, as presented in supplementary Fig. 4d–f.

These findings suggest that while most chromatin modification regions and safe harbor regions are initially preferred by HIV for integration during the acute phase, they tend to be less stable over the course of ART. This instability results in a shift, with integration sites increasingly persisting within in-gene and enhancer regions (Fig. 3j).

### Monoclonal UIS accumulation preferentially at HIV proviral integration sites within in-gene regions in PLWH who initiate ART during the AIDS stage

Monoclonal UIS arise when HIV DNA integrates into specific genomic regions, leading to the expansion of infected cells that harbor these IS. This process is particularly pronounced when the virus preferentially integrates into actively transcribed regions, which are favorable due to their accessibility and high transcriptional activity. The distribution of these proviruses in HIV-infected individuals on long-term ART is shaped by initial integration patterns and selective pressures that favor the survival and clonal expansion of cells with these advantageous integration sites, resulting in the accumulation of monoclonal UIS in-gene regions.^6,27,33,34^

**Fig. 4:**
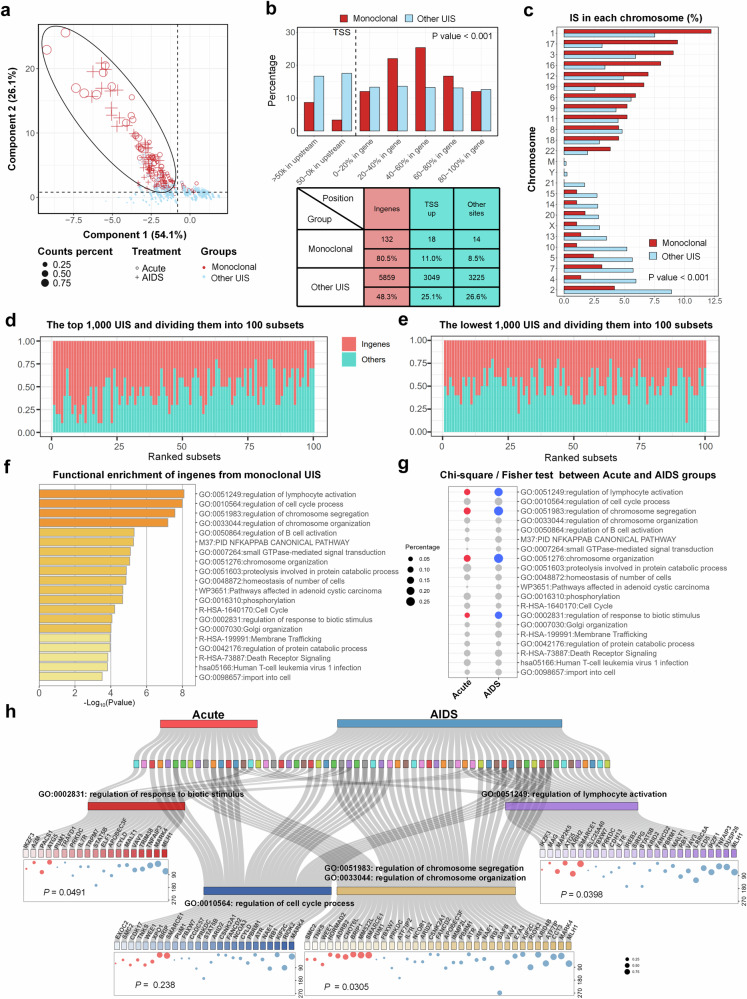
Monoclonal UIS accumulation preferentially occurs at HIV proviral integration sites within in-gene regions during the AIDS treatment stage, with functional enrichment analysis revealing the involvement of these host cell genes in key biological processes. **a** PCA plots distinguishing monoclonal UIS (red dot, *n* = 164) from other UIS (blue dot), the cluster based on LTR percentage, Richness, Evenness and PMD. The plot shows clear distinction between monoclonal UIS with other UIS by two principal components (Component 1, 26.1%; Component 2, 54.1%). **b** Comparison of UIS clonality distribution across different genomic regions (*x*-axis), indicating that monoclonal UIS (red bars) are more likely to integrate into in-genes (Dashed line, right side) than non-monoclonal UIS (blue bars) are more likely to integrate into transcription start site (TSS) upstream (Dashed line, left side) (up panel). The accompanying table indicates that the sites of most monoclonal UIS, 80.5% (132) of integrations occur in in-gene regions, 11.0%^18^ of integrations occur in TSS upstream regions, 8.5%^14^ of integrations occur in other sites. In contrast, other clonal types, 48.3% (5859) of integrations occur in in-gene regions, with 25.1% (3049) in upstream promoter regions, with 26.6% (3225) in other sites (below panel). **c** Chromosomal distribution of monoclonal UIS, highlighting a higher frequency of integration into chromosomes 1, 3, 16, 17, 12, and 19 in monoclonal UIS (red bar) and chromosomes 2, 4, 5, 7, 10, 13, 14, 15, 21, and Y in other UIS (blue bar). **d**, **e** Ranking analysis of all UIS, grouped into sets of 100. Ranking the top 1000 UIS and dividing them into 100 subsets, each containing 10 UIS further organized into subsets of 100. The percentage of UIS integrated into in-gene regions (red) was higher in the top-ranked subset and gradually decreased across the ranks (**d**). In the lowest 1000 UIS, nearly 50% were integrated into genes across the ranks (**e**). **f** Functional enrichment analysis of in-gene regions from 164 monoclonal UIS, highlighting genes enriched in the top 20 biological processes. The x-axis represents-log_10_(*p-value*), with larger values indicating higher statistical significance of enrichment. **g** Chi-square/Fisher test comparing UIS genes percentage in enriched pathways between the Acute- and AIDS-ART groups, showing differences in the percentage of integrated genes across 20 enriched pathways. Blue dots represent pathways with significant differences in the AIDS-ART group compared to the Acute-ART group, indicated by red dots. Dot size reflects the percentage of integrated genes from each pathway in two groups, with larger dots indicating higher percentages. **h** Sankey bubble plot for GO-term classification. This chart illustrates enriched genes associated with lymphocyte activation (GO: 0051249), chromosome segregation (GO: 0051983), chromosome organization (GO: 0033044), regulation of cell cycle process (GO: 0010564), and response to biotic stimulus (GO: 0002831) for two groups—PLWH who initiated ART during the acute stage (red) and those who initiated ART during the AIDS stage (blue). Bubble size represents the UIS count percentage relative to each group, and the *y*-axis indicates ART duration (in months). (Note: *p* < 0.05 indicates statistical significance; Kruskal-Wallis test for (**b**, **c**) Chi-square / Fisher’s exact test for (**g**, **h**) the terms ‘Acute’ and ‘AIDS’ in all figures refer to PLWH who initiated ART during the acute stage (also referred to as the Acute-ART group) and those who initiated ART during the AIDS stage (also referred to as the AIDS-ART group), respectively)

Principal Component Analysis (PCA) plots revealed that monoclonal UIS could be distinctly separated from other UIS, regardless of whether treatment was initiated during the acute phase or the AIDS stage (Fig. 4a). Comparing different genomic regions between the two groups, it was found that monoclonal UIS were more likely to integrate into in-gene regions than non-monoclonal UIS (Fig. 4b). Furthermore, monoclonal UIS were predominantly located in euchromatin, with higher frequencies observed on chromosomes 1, 17, 3, 16, 12, and 19. In contrast, IS and monoclonal UIS in heterochromatin were less frequent compared to euchromatin. The Y chromosome exhibited the fewest IS, with monoclonal UIS being the least prevalent across all chromatin types. On the X chromosome, monoclonal UIS were less common than non-monoclonal UIS (Fig. 4c).

Further investigation involved ranking the top 1000 UIS and grouping them into sets of 100, each containing 10 UIS. The analysis indicated that the percentage of UIS integrated into genes was highest in the top-ranked set and gradually decreased across the ranks (Fig. 4d). Even among the lowest 1000 UIS, nearly 50% were integrated into genes, with no significant difference observed across the ranks (Fig. 4e). Interestingly, PCA plots suggested that monoclonal UIS did not dramatically differentiate between patients who initiate ART early and those AIDS patients who started ART late (supplementary Fig. 5a). However, some early ART patients in the acute phase with <12 months of treatment, located in the left and bottom parts of the PCA plot, are noteworthy. We will focus on these cases and conduct further gene functional analysis in the next phase of our research. Additionally, no significant differences were found in the genome regions among the monoclonal UIS from two groups (supplementary Fig. 5b). These findings underscore that in PLWH initiating ART during the late stage, monoclonal UIS preferentially accumulate in in-gene regions, suggesting a selective advantage for cells with these integration sites. This selective accumulation may contribute to the persistence of HIV-infected cells, underscoring the challenges of eradicating the virus despite prolonged ART.

### Monoclonal HIV integration sites in host cell genes are enriched in lymphocyte activation, chromosome modification pathways with higher integration rates in the AIDS-ART group

Despite ART, some HIV proviral DNA integrated into long-lived memory CD4 + T cells, forming a latent reservoir that is difficult to eradicate. Transcriptionally active gene regions, provide a favorable environment for HIV to hijack the host’s machinery, ensuring efficient viral replication. Notably, monoclonal HIV proviral DNA is enriched in genes involved in lymphocyte activation, cell cycle regulation, and chromatin modification pathways (Fig. 4f, supplementary Fig. 5c). In Fig. 4g, we conducted chi-square and Fisher tests to compare the percentage of integrated genes across 20 enriched pathways between the Acute- and AIDS-ART groups. The results revealed a significantly higher number of integrated genes in the AIDS-ART group compared to the Acute-ART group (*p* < 0.05) for Gene Ontology (GO) terms such as regulation of lymphocyte activation, chromosome segregation, chromosome organization, and response to biotic stimulus. However, no significant difference was observed for the GO term related to cell cycle regulation, death receptor signaling and the related pathways.

In Fig. 4h, we display all monoclonal integrated genes from both the Acute- and AIDS-ART groups, focusing on those enriched in GO terms for the regulation of lymphocyte activation, chromosome segregation, chromosome organization, response to biotic stimulus, and cell cycle regulation. We also represent clonal size as bubbles with treatment duration to direct readers’ attention on the relationship between clonal expansion and treatment duration.

We highlight that monoclonal integration, where a single infected cell proliferates into a dominant clone, often involves genes such as *SMARCE1, SMC2, MARK4, ARID2, CYLD*, and *CCDC57*, which regulate infected cell survival and proliferation. These genes, actively transcribed and accessible, exhibit random integration patterns without significant differences between the Acute- and AIDS-ART groups. This integration may disrupt normal cell function and promote uncontrolled proliferation, contributing to the persistence of HIV. HIV also frequently integrates into lymphocyte activation genes, such as *CD5, IL7R, STAT5B, TNFAIP3*, and *MALT1*, which are actively transcribed during immune activation, with higher integration rates in the AIDS group. Furthermore, integration into chromosome modification genes may alter the epigenetic landscape, promoting the survival and proliferation of HIV-infected cells.

### Early monoclonal UIS in Acute-ART group functionally differs from long-term dual monoclonal UIS in AIDS-ART group

**Fig. 5:**
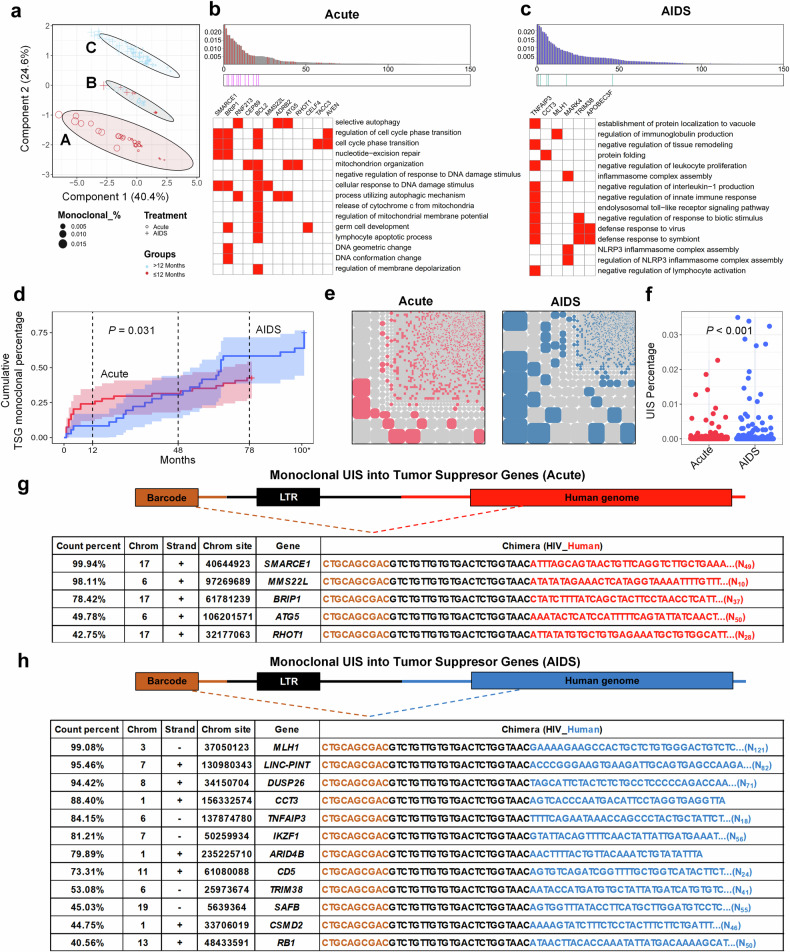
HIV provirus integrated into TSGs is more likely to result in monoclonal expansion in AIDS-ART group, posing a higher cancer risk compared to those in Acute-ART group. **a** PCA plot analysis of monoclonal UIS. The PCA plot highlights differences in monoclonal UIS between Acute-ART group (dots) and AIDS-ART group (crosses). All the monoclonal UIS clustered into 3 group, A, B and C. Blue indicates PLWH with ART duration over 12 months, while red indicates those with ART duration of less than 12 months. **b**, **c** Ranked GSEA of clusters A and C based on clonal normalized size, with similarity analysis on the top 100 enriched pathways, distilled to 15 key pathways and their associated core genes (upper panel). The x-axis represents clonal normalized size, while the y-axis shows the top 100 enriched pathways. The heatmap (lower panel) illustrates the 15 key pathways and corresponding core genes within each pathway for the Acute-ART group (**b**) and the AIDS-ART group (**c**). **d** Cumulative curve of individuals percentage with TSGs monoclonal during ART duration. The cumulative curves displayed the percentage of individuals with TSGs monoclonal (*y*-axis) over time (*x*-axis) in the Acute-ART group (red line) versus AIDS-ART group (blue line), illustrate the preference for HIV integration into TSGs over time. **e** Mosaic chart of TSG monoclonal UIS distribution. This chart illustrates the relative percentage of TSGs monoclonal in two groups—Acute-ART group (left, *n* = 54) and AIDS-ART group (right, *n* = 36). A larger block area indicates a higher prevalence and greater homogeneity of TSGs monoclonal, while a smaller block area reflects increased heterogeneity in clonal distribution patterns. **f** The bar chart shows the comparison of the percentage of TSGs monoclonal among all PLWH from two groups: those initiating ART during the acute phase (red) and those during the AIDS stage (blue). **g**, **h** TSGs integrated by HIV to be monoclonal UIS. List of UIS (count percent < 40%) into TSGs, when disrupted by HIV integration into in-genes region and subjected to clonal expansion, become monoclonal types. This poses a greater cancer risk in Acute-ART group (**g**) compared to AIDS-ART group (**h**). (Note: *p* < 0.05 indicates statistical significance; Wilcoxon test for (**f**); Random linear regression for (**d**); the terms ‘Acute’ and ‘AIDS’ in all figures refer to PLWH who initiated ART during the acute stage (also referred to as the Acute-ART group) and those who initiated ART during the AIDS stage (also referred to as the AIDS-ART group), respectively)

We clustered all patients with monoclonal UIS based on Evenness and Richness data, incorporating ART duration to rank them in a PCA plot. This analysis highlighted distinct patterns of UIS between the two groups, with 164 monoclonal UIS clustering into three distinct groups (A, B, and C). Notably, the separation of monoclonal UIS from the Acute-ART group (Cluster A) with treatment durations of <12 months from those in the AIDS ART group (Cluster C) with treatment durations exceeding 12 months (Fig. 5a) emphasizes the potential influence of early ART on the clonal architecture of the HIV reservoir. This distinction likely reflects differences in immune activation, viral replication dynamics, and integration sites preferences between early and late ART initiation.

To further refine our understanding, we conducted a ranked gene set enrichment analysis (ranked GSEA) on Clusters A and C, using clonal normalized size as a ranking factor. We then conducted a similarity analysis on the top 100 enriched pathways, which we distilled into 15 key pathways with their associated core genes. The results indicate that core genes in the Acute-ART group include *BCL2* (enriched in 11 of 15 pathways), which is involved in mitochondrial-related apoptosis; *BRIP1* (enriched in 7 pathways), related to DNA conformation and cell cycle regulation; and *SMARCE1* (enriched in 4 pathways) (Fig. 5b). In contrast, core genes in the AIDS-ART group include *TNFAIP3* (enriched in 10 pathways), associated with the negative regulation of lymphocyte activation; *TRIM38* (enriched in 3 pathways), linked to the negative regulation of innate immune response; *MAPK4* (enriched in 3 pathways), related to inflammasome complex assembly; and *APOBEC3F* (enriched in 2 pathways), associated with defense responses to viruses (Fig. 5b). An ordinary gene enrichment analysis of the three identified clusters was also conducted, with results available in the supplementary section. (supplementary Fig. 5d).

### HIV provirus integrated into TSGs is more likely to result in monoclonal expansion in AIDS-ART group, posing a higher cancer risk compared to those in Acute-ART group

The integration of HIV proviruses into host genes, particularly TSGs, can significantly affect cellular functions, with profound implications for cancer development.^24–30^ HIV frequently integrates into specific genes as monoclonal types, which can act as risk factors for tumorigenesis. TSGs play a critical role in regulating cell growth and division, serving as a defense mechanism against uncontrolled cell proliferation.^35–37^ However, when HIV integrates into TSGs, it can disrupt their normal function, leading to a loss of regulatory control and increasing the risk of unchecked cell growth and cancer.^38,39^ Therefore, evaluating the tumorigenic potential of monoclonal UIS is crucial. We identified TSGs by cross-referencing several databases, including TSGene2.0, COSMIC, Cancermine, NCG (Network Cancer Genes), and OncoKB, which together provided a list of 3,973 genes. We then compared this list to our UIS data, identifying 14 TSGs in the Acute-ART group and 34 TSGs in the AIDS-ART group (Supplementary Fig. 6a). The cumulative percentage of HIV-integrated TSGs indicates that PLWH who initiated ART during the AIDS stage have a higher prevalence of TSG-integrated monoclonal UIS than those who initiated ART during the acute stage (Fig. 5d).

In PLWH who initiate ART during the AIDS stage, HIV provirus more frequently integrates into specific genes as monoclonal types, significantly increasing the cancer risk compared to those who start ART during the acute phase (Fig. 5e, f). This risk is further compounded by clonal expansion, where cells harboring proviruses integrated into TSGs or other critical regulatory genes undergo proliferation, resulting in an accumulation of cells with oncogenic potential. To illustrate this, we listed top UIS with count percentages >40%, where clonal expansion of TSG-integrated sites contributes to monoclonal expansion, posing a heightened cancer risk in PLWH initiating ART during the AIDS stage, compared to those treated during the acute phase (Fig. 5g, h). We list the remaining UIS associated with TSGs in supplementary Fig. 6b, c. Collectively, these findings underscore the critical importance of early ART in preventing the clonal expansion of infected cells, reducing the formation of stable and persistent reservoirs, and ultimately overcoming one of the major obstacles to achieving a functional cure for AIDS.

## Discussion

In this study, we present a sequencing method for assessing provirus size in human peripheral blood, providing evidence that PLWH who initiate ART during the acute stage have lower reservoir levels compared to those starting ART during the AIDS stage. Our findings also reveal a widespread presence of monoclonal distribution of integration sites in PLWH initiating ART during the AIDS stage, which may pose significant barriers to effective treatment.

During the acute phase of HIV infection, certain CD4 + T cells are initially infected by the virus. These cells, due to their activated state and open chromatin configuration, are more susceptible to viral integration.^24–30^ Following ART, the virus is rapidly suppressed to undetectable levels, resulting in fewer new integration events. Over time, the infected cells either diminish or are lost due to the natural lifespan of CD4 + T cells.^14–18^ Despite this, studies show that at least 40% of long-lived, persistently infected cells exist in a small number of clones, each originating from a single infected cell. These clonally expanded cells can persist for extended periods, with some documented to last up to 11 years.^22–24^ This clonal expansion contributes to the accumulation of monoclonal types, forming stable and persistent reservoirs that complicate efforts to eradicate the virus.^6,24–26^

During the AIDS stage, PLWH may harbor a higher viral reservoir compared to those during the acute stage. By the time ART is initiated during the AIDS stage, the virus has had a prolonged period to replicate and integrate, leading to a substantial viral reservoir. Although the total number of UIS from provirus-integrated cells may not be large, the proliferation of monoclonal UIS from primary cells with the same integrated gene sites significantly contributes to a larger viral reservoir in PLWH initiating ART during the AIDS stage. These cells likely undergo host immune selection, resulting in the formation of stable viral reservoirs. This clonal expansion complicates efforts to eradicate the virus, even with effective viral suppression under ART. In contrast, early ART initiation, particularly during the acute phase, delays the accumulation of monoclonal UIS. By significantly reducing the number of viral integration events, early treatment lowers the likelihood of monoclonal accumulation, similar to oncogene-driven clonal expansion in tumors. This delay in clonal expansion limits the establishment of stable and persistent HIV reservoirs, thereby reducing barriers to achieving a functional cure.

The enrichment of proviral integration in genes related to cell cycle and lymphocyte activation pathways is critical for establishing latent reservoirs, consistent with previous findings that HIV-1 integration sites are often located in highly expressed and cancer-related genes. Unlike the earlier study, which attributed the lack of evidence for clonal expansion to an optimized nonrestrictive linear amplification PCR (nrLAM-PCR)’s inability to distinguish identical integration sites from different cells, they also suggested that the low number of amplified HIV-1 integration sites at certain time points and smaller viral reservoirs in individuals who initiated ART during primary HIV-1 infection might have contributed to this.^40^ In contrast, our research found that clonally amplified cells, sharing similar integration sites, are often referred to as monoclonal types. The increasing monoclonality reduces the immune system’s heterogeneity, potentially increasing susceptibility to infections and raising the risk of lymphoma due to the long-lived nature of these cells. This could be due to the larger number of IS analyzed and the inclusion of a more extensive, long-term follow-up cohort in our study.

However, one limitation of our study is that our method does not differentiate between intact and defective proviruses. Our research focuses on host genes with integrated proviruses and UIS, treating each as a clonal type originating from a single integration event. Cells harboring intact proviruses have the potential to activate viral release or protein expression, making them suitable targets for “shock and kill” strategies aimed at viral clearance. Conversely, surviving cells with defective proviruses—particularly those expanding as large monoclonal populations—may undergo enhanced proliferation due to host genome alterations, which increases the risk of tumor development. Additionally, virus antigen-specific CD8 + T cells are unable to effectively target these defective provirus-infected cells, which may undergo immune escape or experience heightened immune exhaustion,^41^ further facilitating the survival and clonal expansion of cells harboring defective proviruses. This dynamic poses a significant challenge to achieving a functional cure. In our future research, we plan to address this limitation by integrating proviral intactness evaluation alongside host gene sequencing.

Another limitation of our study is the inability to determine the precise number of HIV-infected cells assayed. Our methodology specifically targets the identification and characterization of host genes at HIV integration sites, rather than quantifying the total number of infected cells. As our methods focus on pinpointing integration sites, it is not feasible to ascertain the exact number of HIV-infected cells analyzed. This limitation should be considered when interpreting our results, as they are based on integration patterns rather than on infection frequency across cell populations.

While ART effectively suppresses viral replication, it does not eliminate the latent reservoir. Cells with proviral integrations in critical pathways are more likely to persist and may reactivate if treatment is interrupted.^14–18^ Approaches such as “shock and kill” aim to reactivate latent proviruses, making them susceptible to immune clearance or antiretroviral drugs. Identifying and targeting cells with proviral integrations in-gene regions could enhance the efficacy of these strategies. Additionally, gene editing technologies like CRISPR/Cas9 are being explored to specifically target and excise proviral DNA from the host genome, including those integrated into in-gene regions. Immunotherapeutic approaches, such as engineered T cells or broadly neutralizing antibodies, also hold promise in enhancing the immune system’s ability to recognize and eliminate cells harboring latent proviruses.^42,43^ However, we emphasize that strategies to target and eliminate these latent reservoirs must account for the preferential IS to enhance the efficacy of treatment approaches.

In summary, the preferential integration of HIV proviral sequences into in-gene regions and the accumulation of monoclonal UIS during AIDS treatment underscore the complexities of HIV persistence. Early intervention plays a crucial role in slowing the clonal expansion of infected cells, reducing the formation of stable and persistent reservoirs, and ultimately lowering the barriers to achieving a functional cure. Understanding the dynamics of clonal expansion and preferential integration sites is essential for developing targeted strategies to achieve the goal of functional cure, eliminate latent reservoirs and improve health outcomes for individuals living with HIV.

## Materials and methods

### Participants and clinical data

This study involved participants aged 20 to 70, all of whom provided informed consent and were recruited from the Third People’s Hospital of Shenzhen, China. The study was approved by the Ethics Committee of the Third People’s Hospital of Shenzhen (Approval No. 2023-095-02) and conducted in accordance with the principles of the Declaration of Helsinki (2013) by the World Medical Association. Written informed consent was obtained from all participants before sample collection. A summary of the clinical profiles of the enrolled participants is presented in supplementary Table 1, with a statistical overview of all parameters provided in supplementary Table 2.

Participants in our study were individuals with a confirmed HIV diagnosis, classified according to the stages of HIV infection outlined in the Chinese Guidelines for Diagnosis and Treatment of Human Immunodeficiency Virus Infection/Acquired Immunodeficiency Syndrome (2024 edition).^8^ The progression from initial HIV infection to the terminal stage is a prolonged and complex process, characterized by a wide variety of HIV-related clinical manifestations at different stages of the disease. Based on clinical presentations after infection, the entire course of HIV infection is divided into three stages: the acute phase, the asymptomatic phase, and the AIDS phase. In our study, we categorized these participants into two groups based on specific criteria:

1. **Acute-ART group** refers to PLWH who initiated ART during the acute stage. Accordingly, the acute stage typically occurs within the first 6 months after HIV infection. During this period, some individuals may develop clinical symptoms related to HIV viremia and acute immune system damage. The most common symptom is fever (observed in 80%), often accompanied by sore throat, diarrhea, rash, joint pain, lymphadenopathy, or neurological symptoms, etc. However, many patients experience only mild symptoms, which generally resolve on their own within 1–3 weeks.
  At this stage, HIV RNA and p24 antigen are detectable in the blood, while HIV antibodies gradually seroconvert from negative to positive within 2–3 weeks. This process is usually accompanied by a transient decline in CD4 + T lymphocyte count, inversion of the CD4 + /CD8 + T lymphocyte ratio, and abnormal immune activation of T lymphocytes. Some patients may also experience mild reductions in white blood cell and platelet counts, as well as abnormal liver function. In some cases, there may be a rapid decline in CD4 + T cell counts, leading to a swift progression to AIDS.
2. **AIDS-ART group** refers to PLWH who initiated ART during the AIDS stage, the terminal phase of HIV infection. At this stage, CD4 + T lymphocyte counts typically fall below 200 cells/µL, or the clinical manifestations at this stage primarily include HIV-related symptoms, as well as the development of multiple opportunistic infections and tumors.

We collected comprehensive clinical data for each PLWH (see supplementary Table 1), along with peripheral blood samples, from which genomic DNA was extracted according to the manufacturer’s instructions (QIAamp DNA Blood Kits; QIAGEN, Germany). Utilizing Linear Target Amplification PCR (LTA-PCR) and Next Generation Sequencing, we conducted a longitudinal examination of proviral integration sites. This analysis compared reservoir levels, clonal types, and the predominant gene functions of HIV insertions in oligoclonal populations between PLWH who initiated ART during the acute stage and those during the AIDS stage. This approach enabled a thorough evaluation of integrated HIV provirus.

### LTA-PCR and next generation sequencing

LTA-PCR is employed to amplify and sequence unknown genomic sequences adjacent to integrated vector DNA. This method has been successfully combined with LAM-PCR^44^ and S-EPTS/LM PCR (Selective expandable target sequence/Linker-Mediated).^45^ In brief, 3 × 500 ng of genomic DNA was sheared to a median length of 400 to 500 bp using the Covaris M220 instrument. The sheared DNA was then purified using AmpureXP beads (Beckman Coulter, Inc., USA), and primer extension was performed with a vector-specific biotinylated primer. The extension product was purified again, followed by magnetic capture of the biotinylated DNA using Dynabeads M280 (Thermo Fisher Scientific Inc., USA) for at least 60 min, with two washing steps in 150 µL of H_2_O. The captured DNA was ligated to linker cassettes containing a molecular barcode (Dakewe Biotech Co., Ltd., China). The ligation product was divided into two aliquots and subjected to a first-round nested PCR using biotinylated vector- and sequencing adapter-specific primers. The biotinylated PCR products were magnetically captured, pooled, washed, and half of the eluate was used as a template for a second-round nested PCR with primers designed for deep sequencing using MiSeq technology (Illumina, Inc., USA) after purification. The preparation for deep sequencing was previously described.^46^ DNA double barcoding was employed to enable the parallel sequencing of multiple samples in a single run while minimizing cross-contamination. The detailed LTA-PCR method was expanded in the supplementary section including Real-Time PCR for HIV-1 DNA Quantification in Blood Cells.

### Computational analysis of HIV provirus integration sites

After acquiring sequencing reads, the Genome Integration Site Analysis Pipeline (GENE-IS)^47^ is employed to determine integration frequencies. This process includes sequence trimming, alignment using UCSC BLAT (BLAST-like Alignment Tool),^48^ and the identification of nearby genes and other genomic features, enabling high-throughput analysis of vector IS in both preclinical and clinical settings. The core approach involves calculating integration frequencies by analyzing the proportion of read counts corresponding to each integration event. The effectiveness of GENE-IS in quantifying IS depends on its ability to accurately identify valid reads originating from these sites. Initially, GENE-IS rigorously filters out reads containing megaprimers and anchor sequences from the edges of the integration vector, ensuring the authenticity of sequences originating from the integration sites.

Subsequently, GENE-IS employs a two-step alignment process for aligning flanking genomic sequences to the reference genome. First, the BWA MEM algorithm is used for alignment. For the subset of candidate sequences aligned to multiple sites, GENE-IS re-aligns them using the BLAT aligner to ensure that each read is placed in its optimal alignment position. Finally, GENE-IS clusters IS that are in close proximity (within 500 bp), allowing for more precise estimation of the read counts for individual UIS.

These three steps ensure that GENE-IS accurately captures and precisely quantifies reads supporting integration sites. Supplementary Fig. 1 provides a detailed diagram of the GENE-IS analysis pipeline.^47^

Integration sites per Sample: supplementary Table 3 provides the number of integration sites per sample, allowing assessment of detection consistency across samples. Comprehensive Integration List: To support transparency, a full list of integration sites with genomic coordinates and annotations is included in supplementary Table 4.

### Monoclonal identification (Richness, Evenness, PMD)

Clonality serves as an indicator to assess the rarity and distribution of proliferating cells within a sample. A sample is considered polyclonal if the cells do not undergo dominant cloning, and oligoclonal if dominant cloning occurs. To avoid contradictory results that can arise from the Shannon and Simpson indices when different values of α are used, this study employed PMD analysis based on Rényi entropies.^32,49^

Diversity within proliferating cells is evaluated using two key dimensions: richness, which refers to the variety of cells at independent integration sites, and evenness, which indicates the homogeneity of the cell population. Monoclonal cell samples are characterized by a clonality measure of zero, reflecting the absence of diversity.

### Integrated site annotation for enhancer, safe harbor, methylation, acetylation distribution in host genome

Integrated site annotation was performed to map enhancers, safe harbor regions, methylation, and acetylation sites within the host genome. Enhancer locations were sourced from the ImmPort database (accessible at https://www.immport.org/shared/genelists, updated July 2020). Safe harbor regions were identified in-house, following the guidelines outlined in a previous study.^50^ Chromosomal annotations for methylation and acetylation were integrated from relevant epigenetic datasets. This comprehensive approach enabled a detailed analysis of the distribution and potential functional implications of these regulatory elements within the host genome.

### Integrated gene enrichment analysis using Metascape

Gene enrichment analysis was conducted using Metascape (version [v3.5.20240101], accessible at https://metascape.org). A curated list of differentially expressed genes was input into the Metascape platform, with the organism of interest set to [specify organism, e.g., *Homo sapiens*]. The analysis encompassed GO terms, KEGG pathways, Reactome pathways, and other relevant databases. Metascape provided enrichment results, including statistically significant *p*-values and enrichment scores. Additionally, protein-protein interaction networks were visualized, and hierarchical clustering was performed to identify related gene clusters. The results were subsequently downloaded and further analyzed to pinpoint key biological processes and pathways implicated in the study.

### The TSGs were retrieved from databases and cross-referenced with the monoclonal UIS data from both groups

3973 TSGs were retrieved from several databases, including TSGene2.0 (available at https://bioinfo.uth.edu/TSGene/), COSMIC (available at https://cancer.sanger.ac.uk/cosmic), Cancermine (available at http://bionlp.bcgsc.ca/cancermine/), NCG (Network Cancer Genes) (available at http://network-cancer-genes.org/), and OncoKB (available at https://www.oncokb.org/).

### Statistical analysis

We used a linear mixed model to account for variability in follow-up timing and to handle repeated measures within individuals, enabling accurate analysis of longitudinal changes. Additionally, other statistical methods were applied throughout the study, including the Wilcoxon test, Kruskal-Wallis test, and Chi-square/Fisher’s exact test. To control multiple comparisons, false discovery rate (FDR) adjustments were applied using the Benjamini-Hochberg (BH) procedure.

## Supplementary information

Supplementary Files

Supplementary Tables

## Data Availability

The raw sequence data reported in this paper have been deposited in the Genome Sequence Archive^51^ in National Genomics Data Center,^52^ China National Center for Bioinformation / Beijing Institute of Genomics, Chinese Academy of Sciences (GSA: HRA009798) that are publicly accessible at https://ngdc.cncb.ac.cn/gsa. All software and packages used were publicly accessible, and this study does not report original codes. Any additional information required to reanalyze the data reported in this paper is available from the lead contact upon request.
