# Supplementary material for: Comparing acute versus AIDS ART initiation on HIV-1 integration sites and clonal expansion
Source: Signal Transduct Target Ther. 2025 Jan 10;10:23. doi: 10.1038/s41392-024-02113-7 (PMC11718275; doi:10.1038/s41392-024-02113-7)
Supplement: Supplementary file 1 — Supplementary Files [file 41392_2024_2113_MOESM1_ESM.docx]

Supplementary Materials for

**Comparing Acute versus AIDS ART initiation: HIV-1 integration sites and clonal expansion**

Jun Wang, Nan Xiao, Zhengnong Zhu, Haiyan Qiao, Fang Zhao, Lukun Zhang, Jizhou Gou, Mengji Lu, Yun He, Hongzhou Lu, Qian Li,

Correspondence to: [liqian19900801@hotmail.com](mailto:liqian19900801@hotmail.com)，[luhongzhou@szsy.sustech.edu.cn](mailto:luhongzhou@szsy.sustech.edu.cn), or yuer-he@163.com

**This file includes:**

Materials and Methods

Supplementary Figures 1-6

Supplementary Tables 4

**Materials and Methods**

**1. LTR real-time PCR for HIV-1 DNA quantitation in blood cells**

PBMC were isolated from whole blood acquired from PHLWs by Ficoll-PaqueTM PLUS (Cytiva, USA, CatLog:17144003). Genomic DNA was extracted using the Blood/Cell/Tissue Genomic DNA Extraction Kit (TIANGEN, China, YDP304), and qPCR reactions were performed using Taq Pro Universal SYBR qPCR Master Mix (Vazyme, China, Q712) under the following condition: 94°C 30s, 40 cycles (94°C 5s, 60°C 30s). PCR amplification using primer specific for the HIV-1 LTR region (Forward: AGACCAGATCTGAGCCTGGGA; Reverse: ACACAACAGACGGGCACACA), GAPDH region (Forward: GATTCCACCCATGGCAAATTC; Reverse: CTGGAAGATGGTGATGGGATT), and Actin region (Forward: CTCCATCCTGGCCTCGCTGT; Reverse: GCTGTCACCTTCACCGTTCC).

**2. GENE-IS analysis pipeline**

To identify high-quality insertion sites, the raw data obtained using established protocols (1,2) underwent stringent filtering and processing through the following steps:

**Step 1: Raw sequencing data quality control and filtering**

**(1) Barcode sequence filtering**: Reads that do not contain the sample-specific barcode sequence are removed.

**(2) Quality trimming**: Reads are trimmed from the end if they have a sequencing quality Phred score ≤20. (Phred score is a logarithmic measure of base-calling accuracy, reflecting the probability of an incorrect base call.)

**(3) Megaprimer and linker cassette sequence verification**: Raw paired-end sequencing reads are excluded if the initial 100 base pairs of the forward reads lack the megaprimer sequence (amplified PCR primers derived from the vector’s border sequences) or if the reverse reads lack the linker cassette sequence.

**(4) Primer and sequence trimming**: Using Skewer (version 0.1.117), primers, linkers, and anchor sequences are trimmed from the remaining reads. Reads shorter than 30 bp after trimming are discarded (3).

**Step 2: Two-step alignment process for identifying clipped reads**

Filtered reads are mapped to the human genome (hg38) using BWA to detect vector integration sites (4). Specifically:

**(1) Identification of vector-genome structures**: Sequences with a “vector-genome” structure are mapped to hg38, where part of the read aligns with the reference genome, and the remainder contains the vector-derived megaprimer sequence. GENE-IS uses these reads to infer vector integration events and their corresponding sites within the human genome.

**(2) Secondary alignment**: For reads with multiple alignment loci on hg38, GENE-IS performs a secondary alignment using BLAT v3 to determine the most accurate integration site coordinate (5).

**Step 3: Integration site identification and clustering**

**(1) Selection of insertion sites**: Genomic locations of mapped reads with clips are determined, and insertion sites supported by at least 3 independent reads are selected as final output.

**(2) Readjustment of genomic positions**: GENE-IS refines the genomic position of each integration event based on supporting reads, their coordinates, and the clonal proportion of each event within the sample.

**Step 4: Patient-level integration event and clonal proportion analysis**

**(1) IS data assessment**: GENE-IS analyzes the count of each integration site (IS) and the

positional relationship between neighboring ISs, providing a semi-quantitative assessment of the cloning status of transduced cells.

**(2) Top ISs identification**: For each sample, a list of the top 10 ISs with the highest counts is generated, sorted from highest to lowest.

**(3) In-depth patient-specific analysis**: GENE-IS performs a detailed analysis of integration events specific to each patient, evaluating clonal proportions, diversity, and genomic regional preferences of ISs. This analysis also includes the proximity of integration sites to cancer-related genes and the identification of common integration sites, offering insights into their distribution, clonal proportions, and potential risks (6-8).

**(4) Chromosomal annotations and genomic regional preferences**

The chromosomal annotations cover genomic regions such as enhancers, safe harbors, and regions marked by methylation and acetylation. The reference databases used for genomic regional preferences are as follows:

**Enhancer locations**: [ImmPort Gene Lists](https://www.immport.org/shared/genelists) (Accessible at: <https://www.immport.org/shared/genelists>; Updated: July 2020);

**Safe harbor locations**: In-house calculation based on rules specified by Aznauryan, E., et al (9);

**CpG island annotations**: [UCSC Genome Browser](https://genome.ucsc.edu/) (Accessible at: <https://genome.ucsc.edu>/);

**Methylation data**: [ENCODE Project](https://www.encodeproject.org/) (Accessible at: https://www.encodeproject.org/);

**Genomic coordinates of genes**: [UCSC Table Browser](https://genome.ucsc.edu/) (Accessible at: <https://genome.ucsc.edu>/).

**Reference**

1. Schmidt, M., et al. High-resolution insertion-site analysis by linear amplification-mediated PCR (LAM-PCR). *Nat. Methods.* **4**, 1051–1057 (2007).

2. Schmidt, M., et al. Detection and direct genomic sequencing of multiple rare unknown flanking DNA in highly complex samples. *Hum. Gene Ther.* **12**, 743–749 (2001).

3. Jiang, H., Lei, R., Ding, S. W., & Zhu, S. Skewer: a fast and accurate adapter trimmer for next-generation sequencing paired-end reads. *BMC bioinformatics.* **15**, 182 (2014).

4. Li, H., & Durbin, R. Fast and accurate short read alignment with Burrows-Wheeler transform. *Bioinformatics (Oxford, England).* **25**, 1754–1760 (2009).

5. Kent W. J. BLAT--the BLAST-like alignment tool.*Genome Res.* **12**, 656–664 (2002).

6. Gabriel, R., et al. Comprehensive genomic access to vector integration in clinical gene therapy. *Nat Med.* **15**, 1431–1436 (2009).

7. Paruzynski, A., et al. Genome-wide high-throughput integrome analyses by nrLAM-PCR and next-generation sequencing. *Nat Protoc.* **5**, 1379–1395 (2010).

8. Afzal, S., Wilkening, S., von Kalle, C., Schmidt, M., & Fronza, R. GENE-IS: Time-Efficient and Accurate Analysis of Viral Integration Events in Large-Scale Gene Therapy Data. *Mol Ther Nucleic Acids.* **6**, 133–139 (2017).

9. Aznauryan, E., et al. Discovery and validation of human genomic safe harbor sites for gene and cell therapies.*Cell Rep Methods.* **2**, 100154 (2022).

**Figures and Figure legends**





**Supplementary Fig. 1. Workflow of LTA-PCR and next generation sequencing.** This figure presents the LTA-PCR workflow for amplifying and sequencing genomic regions adjacent to integrated vector DNA, in combination with Linear Amplification-Mediated PCR and Selective Expandable Target Sequence/Linker-Mediated PCR. In brief, genomic DNA (3 × 500 ng) is sheared to 400–500 bp, purified, and extended using a vector-specific biotinylated primer. The biotinylated DNA is captured with Dynabeads M280, ligated to linker cassettes with a molecular barcode, and undergoes nested PCR and magnetic capture. Final products are sequenced on MiSeq (Illumina) with DNA double barcoding to allow multiplexing and reduce cross-contamination.





**Supplementary Fig. 2.**

**(a, b) Total LTR DNA levels (LTR/GAPDH) (a) and [Log (IS-seq) counts], [Log (UIS) counts]** in PLWH who initiated ART during the acute stage (red dot, n = 54) versus those during the AIDS stage (blue dot, n = 36). The Y-axis represents **LTR DNA levels** (LTR/GAPDH) (a) and Log-transformed counts (b), respectively.

**(c, d) Normalized UIS counts (c) and [Log_10_ (IS-seq counts)] (d)** **over time** in PLWH initiating ART during the acute stage (left panel, n = 54) and AIDS stage (right panel, n = 36). The X-axis represents different ART treatment time groups, and the Y-axis represents Normalized UIS counts (c) and Log_10_-transformed (IS-seq counts) counts. The grey line indicates the trend line, and the grey shaded area represents the 95% confidence interval (CI) distribution.

**(e) Evenness and Richness tendency in PLWH initiating ART during acute stage vs. AIDS stage.** The X-axis represents different ART treatment overtime, and the Y-axis represents the value of Evenness and Richness.

(Note: p < 0.05 indicates statistical significance; Wilcoxon test for f; Random linear regression for d; the terms 'Acute' and 'AIDS' in all figures refer to PLWH who initiated ART during the acute stage (also referred to as the Acute-ART group) and those who initiated ART during the AIDS stage (also referred to as the AIDS-ART group), respectively.)





**Supplementary Fig. 3. Analysis of HIV integration in chromatin modification regions in PLWH initiating ART during acute stage vs. AIDS stage.**

**(a)** A detailed analysis of preference for integration into chromatin modification regions (CPG, H3k4me1, H3k4me3, H3k9me1, H3k9me3, H3k27me3, H3k4ac, H3k9ac, H3k27ac) between PLWH initiating ART during acute stage vs. AIDS stage.

**(b-e) Cumulative preference for HIV integration into chromatin regions over time.** The cumulative curves depict the preference for HIV integration into various chromatin regions over time (x-axis representing 0-100 months). These regions include chromatin modifications (CpG, H3k4, H3k9, H3k27). The comparison is between PLWH initiating ART in the acute phase (red) and those in the AIDS stage (blue). The red and blue line indicates the trend, with the shaded area representing the 95% confidence interval (CI).

(Note: p < 0.05 indicates statistical significance; Wilcoxon test for f; Random linear regression for d; the terms 'Acute' and 'AIDS' in all figures refer to PLWH who initiated ART during the acute stage (also referred to as the Acute-ART group) and those who initiated ART during the AIDS stage (also referred to as the AIDS-ART group), respectively.)





**Supplementary Fig. 4. HIV integration on chromatin regions in PLWH from AIDS-ART vs. Acute-ART.**

**(a) The dot plot maps all UIS within known HIV integration chromatin modification regions,** include chromatin modifications (H3k4me1, H3k4me3, H3k4ac, H3k9ac, H3k27me3, H3k27ac,) over the duration of ART. The comparison is between patients initiating treatment during the acute phase (left panel, red shading) and those in the AIDS stage (right panel, blue shading) (x-axis representing months). The comparison is between patients initiating ART in the acute phase (red) and those in the AIDS stage (blue).

**(b) Mosaic chart of UIS distribution.** This mosaic chart illustrates the relative percentage of each unique integration site (UIS) of chromatin modification regions for patients who initiated ART during the acute phase (left) versus those in the AIDS stage (right).

**(c) Percentage of chromatin modifications** (H3k4me1, H3k4me3, H3k27ac, H3k4ac, H3k9me1, H3k27me3, H3k9ac) in two groups. H3k4me3 and H3K27ac domain as a mark of provirus integrated genes in acute.

**(d, e) Duration of relative monoclonal UIS percentages across in-gene regions (d), enhancers, safe harbor, and CpG sites (e) over ART.** The plots depict HIV proviral integration in different gene regions for PLWH initiating ART during the Acute stage (red, top panel) and the AIDS stage (blue, bottom panel). A p-value < 0.05 indicates statistically significant differences between the two groups.

**(f) Mosaic chart of UIS distribution across gene regions in two groups.** This chart shows the relative percentage of the top 5,000 UIS in two disease groups—Acute-initiated ART and AIDS-stage initiated ART—highlighting relative monoclonal UIS percentages across In-Gene Regions, Safe Harbor, and CpG Sites. The chart emphasizes a higher prevalence and greater homogeneity of monoclonal UIS in the AIDS group, while PLWH initiating ART during the acute stage exhibit greater heterogeneity in clonal distribution patterns.

(Note: p < 0.05 indicates statistical significance; Wilcoxon test for f; Random linear regression for d; the terms 'Acute' and 'AIDS' in all figures refer to PLWH who initiated ART during the acute stage (also referred to as the Acute-ART group) and those who initiated ART during the AIDS stage (also referred to as the AIDS-ART group), respectively.)





**Supplementary Fig. 5.**

**(a) PCA plots display monoclonal UIS** between PLWH who initiated ART during the acute stage (dot) versus AIDS stage (cross), and the plots in blue means ART over 12 months (in blue), the plots in red means ART less than 12 months (in red).

**(b) Bar chart comparing the percentage of UIS counts (y-axis) with the relative distance of their respective HIV integration sites from the TSS (x-axis) in two groups.** Acute-phase ART initiation (red bars) and AIDS-stage ART initiation (blue bars). The dashed line indicates the location of the TSS.

**(c) Monoclonal UIS genes enrichment and interaction network diagram.** Circles represent nodes of different gene enrichment clusters, with larger circles indicating stronger effects**.**

**(d) Gene enrichment analysis of 3 clusters (A, B and C) UIS genes derived from Fig.5a.** In the early ART group with a duration of less than 12 months, the UIS genes (cluster A) were most enriched in pathways related to mRNA splicing via the spliceosome, as well as antigen processing and presentation.

(Note: p < 0.05 indicates statistical significance; Wilcoxon test for f; Random linear regression for d; the terms 'Acute' and 'AIDS' in all figures refer to PLWH who initiated ART during the acute stage (also referred to as the Acute-ART group) and those who initiated ART during the AIDS stage (also referred to as the AIDS-ART group), respectively.)


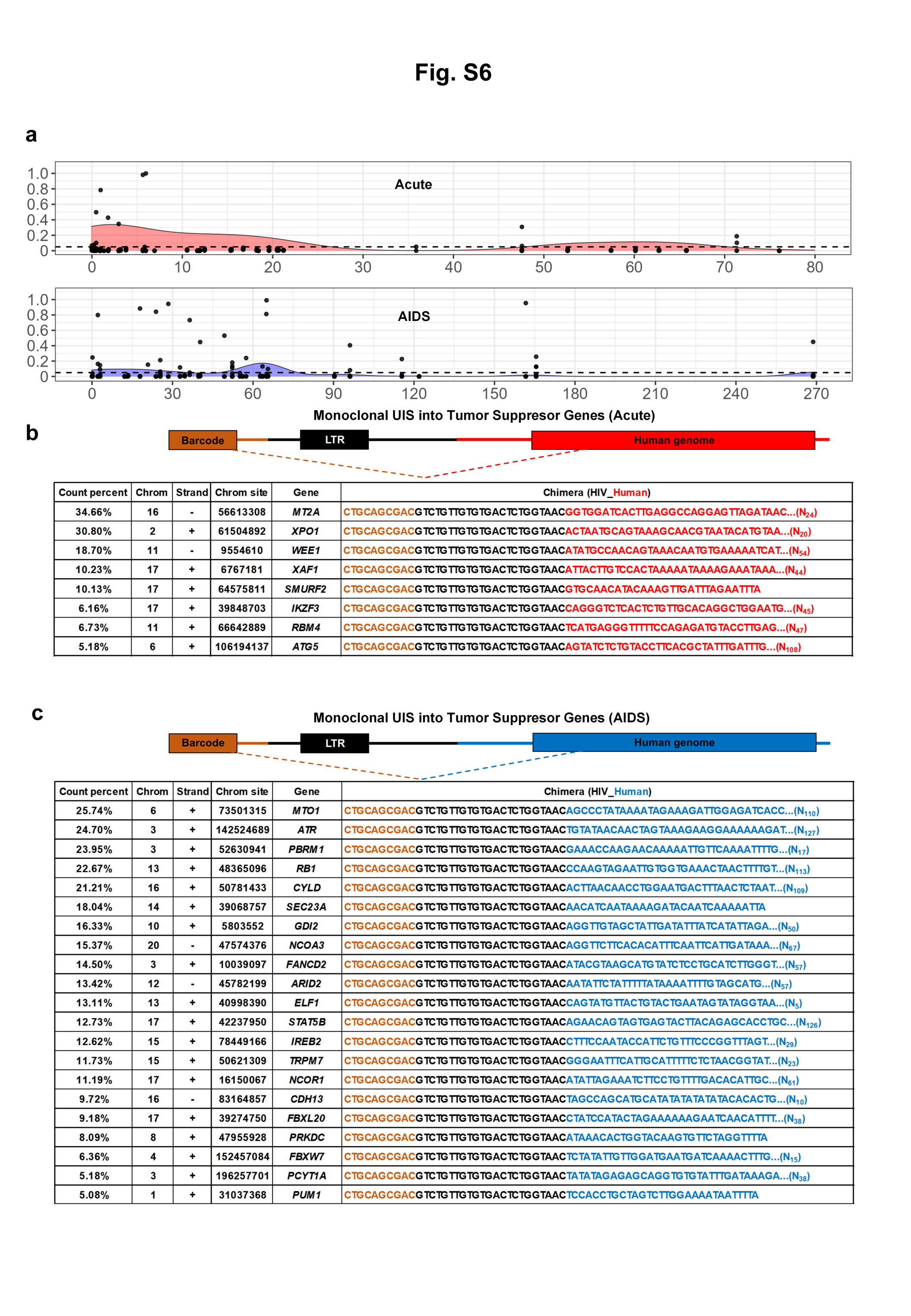


**Figure 6.** **HIV provirus integrated into cancer suppressor genes is more likely to become monoclonal and pose higher risks in AIDS-initiated ART group**

**(a) Duration of monoclonal UIS in Tumor Suppressor Genes (TSGs) over ART.** The plots represent HIV proviral integration into TSGs in the Acute stage ART-initiating PLWH (red, top panel) and the AIDS stage ART-initiating PLWH (blue, bottom panel).

**(b, c) TSG affected by HIV monoclonal UIS.** Tables list the rest of these specific TSGs that, when disrupted by HIV integration into in-genes region and subjected to clonal expansion, become monoclonal types. This poses a greater cancer risk in Acute ART-initiating PLWH **(b)** compared to AIDS ART-initiating PLWH **(c)**.

(Note: p < 0.05 indicates statistical significance; Wilcoxon test for f; Random linear regression for d; the terms 'Acute' and 'AIDS' in all figures refer to PLWH who initiated ART during the acute stage (also referred to as the Acute-ART group) and those who initiated ART during the AIDS stage (also referred to as the AIDS-ART group), respectively.)
